# The Protective Effect of Teprenone on Aspirin-Related Gastric Mucosal Injuries

**DOI:** 10.1155/2019/6532876

**Published:** 2019-06-18

**Authors:** Jing Zhao, Yihong Fan, Wu Ye, Wen Feng, Yue Hu, Lijun Cai, Bin Lu

**Affiliations:** ^1^Department of Gastroenterology, First Affiliated Hospital of Zhejiang Chinese Medical University, 54 Youdian Road, Hangzhou 310006, China; ^2^Key Laboratory of Digestive Pathophysiology of Zhejiang Province, First Affiliated Hospital of Zhejiang Chinese Medical University, Hangzhou, China

## Abstract

**Objective:**

Aspirin usage is associated with increased risk of gastrointestinal bleeding. The present study explored the potential of teprenone, an antiulcerative, in preventing aspirin-related gastric mucosal injuries.

**Methods:**

280 patients with coronary diseases, naïve to aspirin medication, were admitted between 2011 and 2013 at the First Affiliated Hospital of Zhejiang Chinese Medical University and randomized into two groups (*n* = 140). The aspirin group received aspirin enteric-coated tablets 100 mg/day, while the aspirin+teprenone group received teprenone 50 mg 3 times/day along with aspirin. The patients were recorded for gastrointestinal symptoms and gastric mucosal injuries during a follow-up period of 12 months with 3-month intervals.

**Results:**

During the 3-month follow-up, no significant difference was observed in the incidence rate of gastrointestinal symptoms between the two groups (*P* = 0.498). However, the incidence rate of gastrointestinal symptoms was significantly lower in the aspirin+teprenone group than in the aspirin group during the follow-ups at 6 months (*P* = 0.036) and 12 months (*P* = 0.036). The incidence rate of gastric mucosal injuries in the aspirin group was significantly increased at 12 months compared to that at 3 months (*P* = 0.016). The incidence rates at 12 months and cumulative for the entire follow-up period in the aspirin+teprenone group were both significantly lower than those of the aspirin group (*P* = 0.049 and *P* = 0.001, respectively).

**Conclusion:**

Long-term use of low-dose aspirin causes varying degrees of gastric mucosal damages and gastrointestinal symptoms; the severity will increase within a certain range with the extension of medication duration. Teprenone mitigates the gastrointestinal symptoms caused by low-dose aspirin, lowering both the incidence and severity of gastric mucosal injuries and exerting a positive protective effect.

## 1. Introduction

Aspirin (acetylsalicylic acid) can inhibit platelet aggregation by suppressing the production of platelet thromboxane A_2_. Therefore, it serves as an antipyretic analgesic, anti-inflammatory, and antirheumatic agent [1]. For decades, aspirin at a low dose has been used as a secondary agent in the treatment and prevention of cardiovascular anomalies [2, 3]. In addition, the use of aspirin for the primary prevention of cardiovascular disease had demonstrated a significant reduction in major cardiovascular events in the drug-using population. The major benefits in male patients included lowered risks of myocardial infarct, while reduced risks of ischemic strokes were presented in female patients [4, 5]. In addition to the demonstrated benefits of low-dose aspirin in the treatment of cardiovascular diseases, the usage of aspirin has been strongly debated owing to an increased risk of gastrointestinal bleeding [6]. Gastrointestinal symptoms are the most commonly observed adverse effects of aspirin, and long-term usage can lead to gastric mucosal injuries including ulcers and bleeding. However, the removal of aspirin therapy has been associated with a higher mortality rate [7]. Therefore, one of the challenges in clinical practice and framing health policy is to identify the measures for preventing gastric mucosal injuries caused by long-term use of aspirin and the procedures to determine if the benefits outweigh the associated risks [8].

The application of misoprostol, omeprazole, lansoprazole, famotidine, and proton pump inhibitors has been investigated for their efficacy in preventing aspirin-induced gastrointestinal injuries [9–13]. However, the choice of these drugs for preventing the gastrointestinal injuries of patients using aspirin remains controversial. According to the recent guidelines [14], PPIs are recommended for patients with high gastrointestinal (GI) risk who could not avoid using nonsteroidal anti-inflammatory drugs (NSAIDs). For patients at low GI risk, PPIs are not recommended, considering its potential adverse effects, high expense, and overuse in clinical settings [15]. Currently, there is paucity in therapeutics investigated for the prevention of aspirin-induced gastrointestinal complications for these low-GI-risk patients who are not recommended to use PPIs [16]. Thus, there is an urgent need for several randomized control trials and observational studies to investigate the efficacy of additional drugs that could be used by these patients in the prevention of aspirin-associated complications.

One of the possible therapeutics to reduce aspirin-induced bleeding could be the usage of an antiulcerative. Teprenone (6,10,14,18-tetramethyl-5,9,13,17-nonadeca-tetraene-2-one), an antiulcerative, exerts a protective effect on gastric mucosal injuries by promoting gastric mucus secretion, cell regeneration, and increased gastric blood flow [17–19] and is reported to be the most common gastromucoprotective agent in clinical usage with a low incidence of side effects [20]. Moreover, our previous study has also shown that teprenone can reduce NSAID-related acute gastric and small intestinal mucosal injuries in rats [21, 22]. Therefore, this study investigated the potential of teprenone in the protection against aspirin-induced gastric mucosal injuries in patients with a long-term routine-dose usage of aspirin.

## 2. Materials and Methods

### 2.1. Study Subjects

A total of 280 patients with coronary diseases who were naïve to routine-dose aspirin and admitted at the outpatient department of the First Affiliated Hospital of Zhejiang Chinese Medical University between 2011 and 2013 were enrolled based on the following inclusion and exclusion criteria.

The inclusion criteria included (a) patients who require long-term usage of low-dose (<200 mg/day) aspirin enteric-coated tablets for the prevention or treatment of cardiovascular and cerebrovascular diseases, excluding other combinations; (b) patients without abnormal results of routine blood tests, urinalysis, routine stool test, fecal occult blood test, and liver and kidney function tests; and (c) patients agreeing to sign the informed consent and complying with the study protocol.

Exclusion criteria included (a) patients with endoscopic findings of erosion, bleeding, ulcers, and other mucosal damages prior to the enrollment; (b) patients taking medications of other nonsteroidal anti-inflammatory drugs (NSAIDs), antacids, or bismuth medications 2 weeks before the study or during the study period; (c) patients with histories of upper gastrointestinal surgeries or upper gastrointestinal hemorrhage, or suspicions or evidence of malignancies (alarm symptoms or signs); (d) patients with severe complications of heart, lung, liver, kidney, blood, endocrine, or other diseases that may cause damages to the gastrointestinal tracts; (e) pregnant or lactating women; (f) patients with histories of allergy to the study drugs; (g) patients who participated in other clinical studies within 1 month prior to the study; (h) patients who stopped using or changed the dose of aspirin during the study period; and (i) patients who were unable to maintain regular follow-ups as per the protocol or comply with all the study protocols.

This study was approved by the Ethics Committee of the First Affiliated Hospital of Zhejiang Chinese Medical University (approval number: 2012043), and signed informed consent forms were obtained from all the enrolled patients.

### 2.2. Grouping

**Figure 1:**
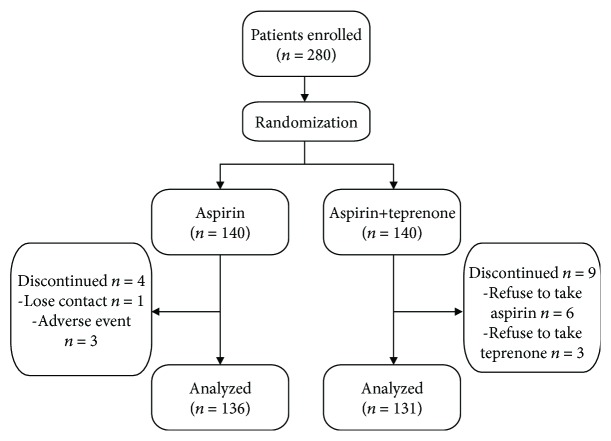
Study flowchart.

Patients were randomized into two groups using the random number table. For the 140 patients in the aspirin group, a daily medication of aspirin enteric-coated tablets (Bayer Pharmaceuticals, Germany), 100 mg, was prescribed, whereas for the other 140 patients in the aspirin+teprenone group, oral medication of teprenone (Eisai Co., Ltd., Japan), 50 mg 3 times/day at 2 h after meals was administered in addition to that of aspirin. The study flowchart is shown in Figure 1.

### 2.3. Data Collection

Details regarding the gastrointestinal symptoms, gastric mucosal injuries, diet habits, alcohol and tobacco habits, presence or absence of family history of gastric cancers, and disease history of the enrolled patients were asked and recorded.

Gastrointestinal symptoms included upper abdominal pain and discomfort, abdominal distension, acid reflux, belching, nausea, changes in stool color, and results of the fecal occult blood test.

Gastric mucosal injuries were assessed based on the clinical manifestations or the gastroscopic findings (the Lanza Endoscopic Scoring System was used to assess endoscopic damage only for those willing to undergo gastroscopy [23]), and data regarding patients' gastric mucosal erosions or ulcers were recorded.

Patients' incidences of gastrointestinal symptoms were recorded every week during the therapeutic period, and the fecal occult blood test was performed at the monthly follow-up.

The three follow-up visiting time points were 3, 6, and 12 months after the medication.

### 2.4. Salvage Therapy

For patients suffering from severe gastric mucosal injuries (especially endoscopic ulcers and bleeding) during the follow-up, esomeprazole 20-40 mg/day and symptomatic treatments were administered. For those with obvious gastrointestinal discomforts, teprenone and other symptomatic treatments were also administered.

### 2.5. Statistical Analysis

Only patients who completed the whole treatment course and all the follow-ups were included in the statistical analysis. The SPSS 19.0 software (IBM, NY, USA) was used for the statistical analysis. Measurement data were expressed as means ± standard deviations [24] and analyzed by the chi-square test. For data that did not match the criteria, the modified chi-square test or Fisher's exact test was adopted. *P* < 0.05 indicated statistical significance.

## 3. Results

### 3.1. General Conditions

In the aspirin group, 136 patients (73 males and 63 females, age: 63.24 ± 9.21 years) completed the study, while in the aspirin+teprenone group, 131 patients (66 males and 65 females, age: 65.73 ± 7.30 years) completed the study. No significant differences were found in age, sex, smoking, alcohol consumption, and diet habit between the groups (all *P* > 0.05, Table 1).

A total of 13 (4.64%) patients from the two groups dropped out from the study at 3 months of medication. Of the 4 drop-out patients in the aspirin group, 1 was lost to follow-up due to changes of contact means and the other 3 were because of skin and mucous ecchymosis. Among the 9 patients from the aspirin+teprenone group, 6 refused to administer aspirin and switched to Chinese patent medicines that can promote blood circulation and the other 3 refused to take teprenone due to excessive combined medications (Figure 1).

### 3.2. Gastrointestinal Symptoms

To test the hypothesis that teprenone mitigates the gastrointestinal symptoms caused by low-dose aspirin, the patients were recorded for gastrointestinal symptoms and gastrointestinal bleeding during a follow-up period of 12 months with 3-month intervals (Table 2).

During the follow-up at 3 months, the gastrointestinal symptoms were mainly manifested as dull upper abdominal pain, discomfort, and abdominal distension. At the 3-month follow-up period, only 2 patients in the aspirin group exhibited gastrointestinal symptoms, while there were none in the aspirin+teprenone group. During the follow-up at 6 months, the gastrointestinal symptoms were mainly manifested as upper abdominal pain, fullness, and acid reflux. During the 6-month follow-up among the aspirin group, 1 patient experienced melena, and 2 patients were positive for the fecal occult blood test. However, none of the patients from the aspirin+teprenone group were positive for the fecal occult blood test. As compared to the aspirin group, the aspirin+teprenone group exhibited a significantly lower (*P* < 0.05) number of patients with gastrointestinal symptoms for the 6-month follow-up period. During the follow-up at 12 months, the gastrointestinal symptoms were mainly manifested as dull upper abdominal pain, belching, and abdominal distension, while other symptoms such as acid reflux and heartburn were also present. Among the aspirin group, 4 patients experienced melena, and 5 patients were positive for the fecal occult blood test. However, only 1 patient from the aspirin+teprenone group was positive for the fecal occult blood test. As compared to the aspirin group, the aspirin+teprenone group exhibited a significantly lower (*P* < 0.001) number of patients with gastrointestinal symptoms for the 12-month follow-up period.

The overall incidence rate of gastrointestinal bleeding was 5.88% in the aspirin group and 1.52% in the aspirin+teprenone group. In addition, the incidence rate of gastrointestinal symptoms in the aspirin group at 12 months increased significantly as compared to those at 3 and 6 months (20.59 vs. 1.47%, *P* < 0.001 and 20.59 vs. 6.62%, *P* = 0.001, respectively). With respect to the cumulative incidence rate for the entire follow-up period, the aspirin+teprenone group exhibited a significantly lower (*P* < 0.001) number of patients with gastrointestinal symptoms.

All patients with gastrointestinal symptoms were relieved after the oral medication of esomeprazole and symptomatic treatments.

### 3.3. Gastroscopy

The findings of the gastroscopy examination for the aspirin and aspirin+teprenone groups during the follow-up are summarized in Table 3.

During the follow-up at 3 months after medication, a total of 57 patients underwent gastroscopy, including 31 patients from the aspirin group and 26 patients from the aspirin+teprenone group. The gastroscopic findings from the aspirin group indicated 7 cases of gastric mucosal erosions and 2 cases of peptic ulcer (1 with multiple ulcers at the gastric antrum and 1 with duodenal ulcer), whereas the gastroscopic findings from the aspirin+teprenone group showed only 2 cases of gastric mucosal erosions. However, no significant differences (*P* > 0.05) were observed in the incidence rates of gastric mucosal injuries between the two groups.

During the follow-up at 6 months after medication, a total of 27 patients received gastroscopy, including 18 patients from the aspirin group and 9 patients from the aspirin+teprenone group. The gastroscopic findings from the aspirin group indicated 4 cases of discrete multiple erosions and 3 cases of ulcers at the gastric antrum, whereas the gastroscopic findings from the aspirin+teprenone group showed only 1 case of discrete multiple erosions at the gastric antrum. However, no significant (*P* > 0.05) difference was observed in the incidence rates of gastric mucosal injuries between the two groups.

During the follow-up at 12 months after medication, a total of 53 patients underwent gastroscopy, including 36 patients from the aspirin group and 17 patients from the aspirin+teprenone group. The gastroscopic findings from the aspirin group indicated 8 cases of gastric mucosal erosions and 13 cases of peptic ulcer (6 with gastric ulcers, 3 with duodenal ulcers, and 4 with compound ulcers), whereas the gastroscopic findings from the aspirin+teprenone group showed 4 cases of gastric mucosal erosions and only 1 case of superficial ulcer at the gastric antrum. The incidence rate of gastric mucosal injuries in the aspirin+teprenone group was significantly lower (*P* < 0.05) than that in the aspirin group.

Cumulatively, 137 patients underwent gastroscopy, including 85 patients from the aspirin group and 52 patients from the aspirin+teprenone group. In the aspirin group, the incidence rate of gastric mucosal injuries was 43.5%, which included 21.2% (*n* = 18) for gastric and duodenal ulcers and 22.3% (*n* = 19) for gastric and duodenal mucosal erosions. On the other hand, in the aspirin+teprenone group, the incidence rate of gastric mucosal injuries was 15.4%, which included 1.9% (*n* = 1) for gastric ulcer and 13.5% (*n* = 7) for gastric mucosal erosions. Compared to the aspirin group, the incidence rate of gastric mucosal injuries in the aspirin+teprenone group was significantly lower (*P* < 0.05) and the severity was also milder.

In addition, as observed from Table 2, the incidence rate of gastric mucosal injuries in the aspirin group at the 12-month follow-up was significantly increased compared to that of the 3-month follow-up (58.46% vs. 29.03%, *P* = 0.049).

## 4. Discussion

Gastrointestinal adverse events are the common effects of aspirin, of which peptic ulcers, bleeding, and perforations are extremely severe. Meanwhile, the protective use of proton pump inhibitors for aspirin-related gastric mucosal injuries is limited, especially to some elderly patients who receive multiple drugs and have multiple underlying diseases. According to current guidelines [25], no specific recommendations have been given for this group of patients, so the use of gastric mucosal protectors with fewer side effects may be a potential choice. In this study, we demonstrate that teprenone, a gastric mucosal protector, ameliorates aspirin-induced gastrointestinal adverse events.

The long-term use of low-dose aspirin can lead to symptoms such as upper abdominal discomforts, nausea, vomiting, and melena, accompanied by the endoscopic manifestations of gastric mucosal erythema, punctate bleeding, erosion, or ulceration [26–28]. Therefore, to investigate the protective effects of teprenone on aspirin-induced gastrointestinal complications, the patients were assessed for gastrointestinal symptoms and gastric mucosal injuries as described in the Materials and Methods during a follow-up period of 12 months at 3-month intervals. In the current study, we found that the long-term usage of low-dose aspirin could cause gastrointestinal symptoms (mainly manifested as dull upper abdominal pain and distention) and induce gastric mucosal injuries as evidenced by the gastroscopic findings. A similar study by Yeomans et al., investigating the adverse effects of aspirin, showed that the incidence rates of ulcer and mucosal erosion in patients due to the long-term usage of low-dose aspirin were as high as 10.7% and 63.1%, respectively [24]. In another study involving over 27000 patients with long-term usage of low-dose aspirin, the findings suggested that the incidence rate of gastrointestinal bleeding was 2.6% [29]. In recent years, studies on the impacts of low-dose aspirin upon patients' gastrointestinal adverse events have been increasingly documented and reviewed [30].

In the current study, during the 12-month follow-up, it was found that the incidence rates of gastrointestinal symptoms and mucosal injuries were positively correlated with the medication duration. This finding is in agreement with the previous studies that clearly show low-dose aspirin causing gastrointestinal symptoms and gastric mucosal injuries, and these adverse effects aggravate with time [30].

In the current situation, the physicians lack the understanding and awareness of the potential risk of gastrointestinal injuries associated with the long-term use of low-dose aspirin. This is because the mechanisms of gastric mucosal injuries caused by the nonsteroidal anti-inflammatory drug (NASIDs) are still unclear. An investigation conducted by Zhu et al. showed that only 3.5% of the physicians would prescribe proton pump inhibitors (PPIs), histamine 2-receptor antagonists (H2RA), and mucoprotective drugs (MPs) for long-term users of low-dose aspirin [29]. It is reported that the use of MPs, PPIs, or H2RA in combination with aspirin can reduce the gastric mucosal injuries [20, 31, 32]. One of the plausible explanations for aspirin-induced gastrointestinal complications is due to the blockage in the synthesis of prostaglandins that subsequently lead to the damage of gastric mucosa [33, 34]. Teprenone, an MP that promotes the endogenous synthesis of prostaglandins (PG), can serve as a broad-spectrum antiulcer agent and has a remarkable ability to repair and restore gastric mucosal integrity by slowing down the rate of its erosion and by stimulating mucus elaboration [17, 35, 36]. Current studies show that teprenone is effective in protecting against gastric mucosal injuries induced by various causes [37, 38]. Our study indicates that the incidence rates of gastrointestinal symptoms in the aspirin+teprenone group both at the 6- and 12-month follow-ups are significantly lower than those of the aspirin group. Also, the incidence rates of gastric mucosal injuries in the aspirin+teprenone group at the 12-month follow-up and cumulative for the entire follow-up were significantly lower than those of the aspirin group. In addition, the gastroscopic scores of the aspirin+teprenone group at 3 months and 12 months, and the cumulative score for the entire follow-up were significantly lower than those of the aspirin group. The data obtained from the present study indicate that teprenone can reduce the gastrointestinal symptoms, lower the incidence rate of gastric mucosal injuries, and to some extent alleviate the severity of gastric mucosal injuries caused by the use of low-dose aspirin.

The current study has a few limitations. The assessment of gastric mucosal injury is a vital index parameter, but only a portion of the patients underwent endoscopy. Therefore, patients should be encouraged to undergo endoscopic examination to evaluate gastric mucosal injury. Moreover, there is a need to conduct a large scale study to replicate our results, assess long-term benefits, and facilitate a full economic evaluation. Furthermore, the study was a single-center study that may have affected outcomes due to the integrity and authenticity of collected data.

In conclusion, comparisons of gastrointestinal symptoms and gastroscopic findings between the two groups have demonstrated varying degrees of gastric mucosal injuries and gastrointestinal symptoms caused by the long-term usage of low-dose aspirin; the severity of these adverse events will aggravate within a specific duration. Herein, the study demonstrated the potential of teprenone as a protective agent for aspirin-induced gastric mucosal injuries, thereby suggesting its clinical application.

## Figures and Tables

**Table 1:** Baseline characteristics of patients.

	Aspirin (*n* = 136)	Aspirin+teprenone (*n* = 131)	*P* value
Male sex, *n* (%)	73 (53.7)	66 (50.4)	0.590
Mean age ± SD (years)	67.24 ± 9.21	65.73 ± 10.30	0.207
Current smoking, *n* (%)	29 (21.3)	25 (19.1)	0.649
Current alcohol consumption, *n* (%)	27 (19.9)	24 (18.3)	0.750
Pickled products, *n* (%)	28 (20.6)	27 (20.6)	0.996
Spicy food, *n* (%)	14 (10.3)	17 (13.0)	0.494

**Table 2:** Gastrointestinal symptoms and bleeding in patients.

	Aspirin (*n* = 136)	Aspirin+teprenone (*n* = 31)	*P* value
3-Month gastrointestinal symptoms, *n* (%)	2 (1.47)	0 (0)	0.494
6-Month gastrointestinal symptoms, *n* (%)	9 (6.62)	2 (1.53)	0.036
12-Month gastrointestinal symptoms, *n* (%)	28 (20.59)	4 (3.05)	0.000
Total gastrointestinal symptoms, *n* (%)	39 (28.68)	6 (4.58)	0.000
3-Month gastrointestinal bleeding, *n* (%)	1 (0.74)	1 (0.76)	0.488
6-Month gastrointestinal bleeding, *n* (%)	2 (1.47)	0 (0)	0.494
12-Month gastrointestinal bleeding, *n* (%)	5 (3.68)	1 (0.76)	0.233
Total gastrointestinal bleeding, *n* (%)	8 (5.88)	2 (1.52)	0.004

**Table 3:** Gastroscopy examination results of patients.

	Aspirin (*n* = 136)	Aspirin+teprenone (*n* = 131)	*P* value
3-Month gastric mucosal erosion, *n* (%)	7/31 (22.58)	2/26 (7.69)	0.242
6-Month gastric mucosal erosion, *n* (%)	4/18 (22.22)	1/9 (11.11)	0.861
12-Month gastric mucosal erosion, *n* (%)	8/36 (22.22)	4/17 (23.55)	0.806
Total gastric mucosal erosion, *n* (%)	19/85 (22.35)	7/52 (13.46)	0.198
3-Month peptic ulcer, *n* (%)	2/31 (6.45)	0/26 (0)	0.551
6-Month peptic ulcer, *n* (%)	3/18 (16.67)	0/9 (0)	0.516
12-Month peptic ulcer, *n* (%)	13/36 (36.11)	1/17 (5.88)	0.046
Total months with peptic ulcer, *n* (%)	18/85 (21.18)	1/52 (1.92)	0.002

## Data Availability

The data used to support the findings of this study are available from the corresponding author upon request.
